# How Methodologic Differences Affect Results of Economic Analyses: A Systematic Review of Interferon Gamma Release Assays for the Diagnosis of LTBI

**DOI:** 10.1371/journal.pone.0056044

**Published:** 2013-03-07

**Authors:** Olivia Oxlade, Marcia Pinto, Anete Trajman, Dick Menzies

**Affiliations:** 1 Department of Epidemiology, Harvard School of Public Health, Boston, Massachusetts, United States of America; 2 Department of Research, Fernandes Figueira Institute, FIOCRUZ, Rio de Janeiro, Brazil; 3 Gama Filho University, Rio de Janeiro, Brazil and McGill University, Montreal, Canada; 4 Respiratory Epidemiology & Clinical Research Unit, Montreal Chest Institute, McGill University, Montreal, Canada; University of Michigan, United States of America

## Abstract

**Introduction:**

Cost effectiveness analyses (CEA) can provide useful information on how to invest limited funds, however they are less useful if different analysis of the same intervention provide unclear or contradictory results. The objective of our study was to conduct a systematic review of methodologic aspects of CEA that evaluate Interferon Gamma Release Assays (IGRA) for the detection of Latent Tuberculosis Infection (LTBI), in order to understand how differences affect study results.

**Methods:**

A systematic review of studies was conducted with particular focus on study quality and the variability in inputs used in models used to assess cost-effectiveness. A common decision analysis model of the IGRA versus Tuberculin Skin Test (TST) screening strategy was developed and used to quantify the impact on predicted results of observed differences of model inputs taken from the studies identified.

**Results:**

Thirteen studies were ultimately included in the review. Several specific methodologic issues were identified across studies, including how study inputs were selected, inconsistencies in the costing approach, the utility of the QALY (Quality Adjusted Life Year) as the effectiveness outcome, and how authors choose to present and interpret study results. When the IGRA versus TST test strategies were compared using our common decision analysis model predicted effectiveness largely overlapped.

**Implications:**

Many methodologic issues that contribute to inconsistent results and reduced study quality were identified in studies that assessed the cost-effectiveness of the IGRA test. More specific and relevant guidelines are needed in order to help authors standardize modelling approaches, inputs, assumptions and how results are presented and interpreted.

## Introduction

Global tuberculosis (TB) control is currently facing great opportunities, but also great challenges. Opportunities for improved TB control have increased dramatically over the past decade as the result of greater funding from governments of low and middle income countries (LMICs) and from international donors and funding agencies [1]. At the same time, the number of new tools, particularly in the area of TB diagnostics, has expanded rapidly, providing a wide array of potential technologies for implementation [2]. One of the greatest challenges for governments and donor agencies is to decide where to invest resources to achieve the greatest benefit for the most people.

Economic analyses can provide decision makers with more information on which to base investment decisions, by comparing costs and resulting health benefits of different approaches. Cost Effectiveness Analyses (CEA) are one of the most commonly used economic analyses in published studies [3]. The cost per unit of outcome or health effect of different interventions can be estimated and compared [3]. If CEAs are conducted with rigorous, standardized and transparent methods, results of different analyses should be comparable and help policy makers reach consensus on interventions to be implemented in a particular population or setting [4]. However, if different analyses of the same intervention produce contradictory results, this may heighten confusion and even discredit the value of these analyses.

The area of diagnostics for latent TB infection (LTBI) serves as an excellent example of this phenomenon. Until relatively recently, a single test – the Tuberculin Skin Test (TST) - was the only method to diagnose LTBI. In the past decade, Interferon Gamma Release Assays (IGRAs) have been approved for use for this purpose in many countries, leading to a wave of studies of their accuracy and utility [5], [6]. These have included cost-effectiveness analyses, which have provided seemingly contradictory messages.

In general, systematic reviews are designed to synthesize evidence after careful assessment of the methodological quality of all available relevant studies on a particular topic [4]. For economic analyses in particular, the goal of a systematic review is not to produce statements about whether a particular intervention is cost effective, but rather to summarize what is known from different settings about economic aspects of interventions, as well as to encourage a more transparent and consistent approach to the conduct and reporting of economic analyses [4]. The objective of our study was thus to conduct a systematic review of *methodologic aspects* (study quality, inputs and methodologic approach) of CEA that evaluate IGRA’s for the detection of LTBI, in order to assess if methodologic differences could account for differences in study findings and conclusions. A second objective was to develop a common decision analysis model that could quantify the impact on predicted costs and effectiveness of the observed differences in inputs that were used in the studies identified.

## Methods

### Ethics Statement

An ethics statement was not required for this work.

### Systematic Review

#### Search criteria

We searched for CEA that compared IGRA’s with at least one other test strategy for diagnosing LTBI. Included studies used modeling techniques to make predictions about specific outcomes over time with any analytic horizon. No limits on year of publication, or language were imposed. Predicted outcomes of interest included Quality Adjusted Life Years (QALYs), active TB cases and total costs predicted. Studies were excluded if they: 1) used animal subjects; 2) assessed detection of active disease; 3) were conference abstracts or proceedings; 4) assessed detection of non-tuberculous mycobacterial infection or disease; and 5) used non-standard tests for LTBI.

#### Search methods

We searched the following databases from 1947 up to March 15^th^ 2011: Scopus, Web of Science, Medline, Embase, Cinhal, Cochrane Library, CRD, Econlit, CEA registry and Lilacs for relevant studies. An update was performed on August 31 2011. In addition to these databases, reference lists of identified publications were also hand searched. A sample search string used for a Medline database search can be found in Table S1.

#### Study selection

Two independent reviewers reviewed all titles and abstracts in order to select full studies. Full text review to finalize study selection was done independently by the same two reviewers and any disagreements were resolved by a third reviewer.

#### Data abstraction

A standardized data abstraction form was developed and piloted on a subset of studies. Once finalized, the form was used by two reviewers to independently extract data. Data was extracted on the following topics; 1) General information, 2) Model/Economic inputs and assumptions, 3) Study input data sources, 4) Predicted outcomes and 5) Study quality (using the Drummond Checklist- see section below for more detail). More detail on the types of data abstracted by study can be found in Table S2. Data from both reviewers was compared to ensure accuracy of data abstraction. Any differences between reviewers were resolved by discussion with a third reviewer. Authors were contacted for clarification or if key information was missing.

#### Assessment of study quality

For each study, the overall methodological quality of each study was evaluated using the Drummond et al. 35 item checklist [7]. Each individual item was scored using the mutually exclusive categories “Yes”, “No”, “Not clear” or “Not applicable”. A detailed qualitative comparison of the results provided in the text and abstract conclusion was also conducted.

### Summarizing Variability in Study Inputs and Predicted Results

For each study the following model inputs were abstracted: test characteristics, transitional probabilities (eg risk of disease if infected), and costs - particularly the specific components of the cost for IGRA and TST. Predicted outcomes abstracted included: cost per person screened and effectiveness measures (QALYs or active cases) by test scenario. All costs were converted to US dollars [8]–[11] and adjusted for inflation to 2011 US dollars [12].

### Assessment of Impact of Variability in Study Inputs using a Common Model

#### Decision analysis model

We developed a common decision analysis Markov model using TreeAge software (TreeAge, Version 2011) that incorporated the basic structure and consequences of all of the models used in the studies included in the review. As shown in Figure S1, the model simulates two identical population cohorts, some of whom are infected with TB. In the first year of the simulation, the first cohort is tested with an IGRA test, while the second is tested with a TST. Depending on the underlying TB health state of the population, and the characteristics of the test being used, the population falls into one of four mutually exclusive states (true positive, false positive, true negative or false negative). Depending on the state, various consequences ensue. For example, for those who are test positive, some of the population may adhere to treatment and complete an effective course of treatment, resulting in no negative outcome. Non-completion and/or ineffective therapy can also occur however, which results in the development of active TB- a negative predicted outcome. Adverse events can also occur to anyone who is treated, regardless of underlying TB health state. Once the cohort completes the screening and treatment process in the first year of the model, those that are infected and remain with LTBI will cycle into an “infected state” in the next year of the model and may later reactivate and develop active disease. Those who cure after prophylactic therapy, or reactivate to active disease do not continue to cycle in subsequent years.

#### Assessing impact of input variability on predicted results

In this common model all pathogenetic and cost inputs were defined using the distribution of input values used in the different studies included in the review. These cost and input values that affect effectiveness are summarized in Tables 1 and 2. Monte Carlo probabilistic sensitivity analysis was used over 10,000 iterations to define the distribution of outcomes (costs and effectiveness) for each test scenario. Effectiveness was defined as the probability of being an active case. Percentiles (2.5^th^ and 97.5^th^) were calculated for each distribution and predicted results were plotted in order to visually compare results.

**Table 1:** Summary of Key Epidemiologic Inputs of Effectiveness.

Author,Year	InitialPrevalenceof LTBI	AnnualReactivation rate	TSTSensitivity	TST Specificity- NO BCG	TST Specificity-BCG	IGRASensitivity	IGRASpecificity	LTBI Completionrate	Efficacy of Full LTBI regimen	Probability of adverse event
Burgos, 2009	58%	1.10%	NA	NA	NA	95%	98%	80%	69%	18%
de Perio, 2009	5%	0.02%	67%	98%	70%	76%	96%	31%	90%	0.60%
Deuffic-Burban, 2010	41%	0.24%	73%	NA	60%	76%	96%	57%	69%	0.001%
Diel, 2007	11%	0.30%	90%	NA	61%	90%	100%	100%	80%	0%
Diel, 2007	28%	0.30%	93%	NA	25%	95%	100%	100%	80%	0%
Kowada, 2010	5%	0.28%	80%	97%	59%	84%	99%	80%	70%	0.30%
Kowada, 2010	36%	0.15%	NA	NA	NA	81%	99%	80%	70%	0.30%
Kowada, 2008	20%	0.28%	71%	98%	15%	76%	96%	90%	70%	1.30%
Linas, 2011 (adult close contacts)	43.7%	1.02%	89%	98%	NA	83%	99%	48%	90%	1%
Linas, 2011(recent adult immigrants to US)	41.4%	0.08%	89%	NA	92%	83%	99%	51%	90%	1%
Marra, 2008	21%	0.41%	99%	95%	36%	99%	96%	28%	90%	0.30%
Oxlade, 2007	35%	0.10%	95%	98%	60%	95%	98%	21%	80%	1%
Pareek, 2011	23%	0.25%	NA	NA	NA	84%	99%	81%	65%	0.20%
Pooran, 2010	30%	1.25%	85	80%*	NA	95%/89%**	100%/95%**	72%	65%	0.3%

More detail provided in Table S1.

* BCG status of population not specified.

** T Spot/IGRA.

**Table 2:** Key cost components extracted from studies included in review (Adjusted to 2011 USD).*

Author,Year	Full LTBI tx cost	Adverse event cost	Active TB cost	TST test cost	IGRA test cost
Burgos, 2009	$264**	$272	$11,271	N/A	$21
de Perio, 2009	$540	$338†	$63,120	$60	$49
Deuffic-Burban, 2010	$498	$6,814	$8,308	$17	$71
Diel, 2007	$299	N/A	$28,007	$32	$79
Diel, 2007	$1,577	N/A	$24,679	$39	$219
Kowada, 2010	$910	$14,006	$17,508	$109	$117
Kowada, 2010	$574	$13,245	$16,556	N/A	$92
Kowada, 2008	$472	$10,898	$13,623	$121	$118
Linas, 2011	$462	$183	$13,378	$42	$52
Marra, 2008	$462	$698	$17,077	$28	$50
Oxlade, 2007	$512	$6,293	$25,553	$15	$47
Pareek, 2011	$224	$2,344	$5318	N/A	$77
Pooran, 2010	$953	$1143	$13,849	$29	1) T-Spot: $100 2) QFT: $82

* List is not a comprehensive list of all costs included in studies, but is restricted to those costs included in common decision model described in main text.

N/A = Cost not included in study.

Full LTBI tx cost = includes cost for complete regimen cited in publication.

Active TB Cost = cost for a passively diagnosed Active case.

IGRA test cost = All studies considered Quantiferon, except Reference (12) (T-Spot only), Reference (16) (generic IGRA) and Reference (20) (included both QFN and T-spot).

** Cost is for drugs and incentives only. Administrative and delivery expenses associated with LTBI treatment are included in the fixed program costs of 150 US$ (2007) per year per study participant. These additional costs are not accounted for in any of the costs categories listed above.

† Cost shown for mild hepatitis.

One way sensitivity analysis was then conducted on each variable included in this common decision analysis model to quantify the relative impact on predicted total costs over a 20 year analytic horizon of differences in the study inputs. For this analysis, the input range for each variable was taken from the maximum and minimum of values used in the different studies included in the review. The spread (calculated as the difference between the lowest predicted outcome value, and the highest expected value) and potential influence (calculated as the Spread divided by the mean expected value for each test scenario) of each variable was calculated.

## Results

### Studies Included in Review

**Figure 1:**
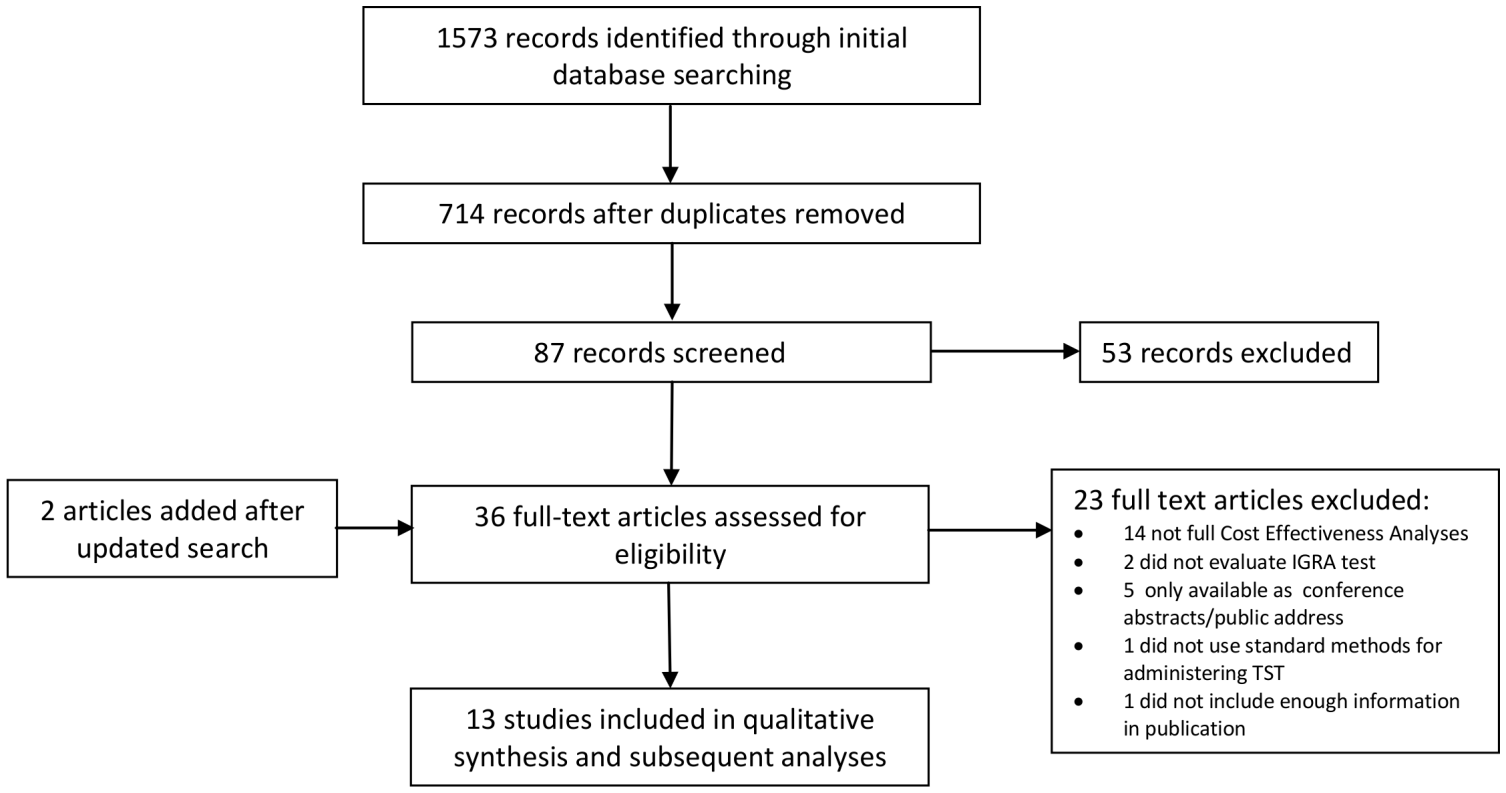
Flow Chart of Study Selection.

As shown in Figure 1, the initial search found 714 unique references. After review of titles, abstracts and full text, 11 studies met the inclusion criteria and were included in the review. Two additional studies published after the initial review was conducted were added when a search update was performed. A summary of the 13 studies included in the review is provided in Table 3 [13]–[25]. Studies mostly considered populations in high income countries. A variety of study sub-populations were considered including contacts, immigrants, health care workers.

**Table 3:** Summary of Studies included in Review.

Authors	Year Published	Population	Mean age at start ofanalysis (Basecase)(yrs)	Reference
Burgos et al.	2009	Mexico- high TB/HIV risk sub population	Adult	(13)
de Perio et al.	2009	US Health Care workers	35	(14)
Deuffic-Burban et al.	2010	France, Contacts	35	(15)
Diel et al.	2007	Germany, Close contacts	20	(16)
Diel et al.	2007	Swiss, Contacts	20 (or 40)	(17)
Kowada et al.	2010	Japan, Rheumatoid Arthritis patients	40	(18)
Kowada et al.	2010	Japan, elderly	65	(19)
Kowada et al.	2008	Japan, contacts	20	(20)
Linas et al.	2011	US, 19 different high risk groups	Risk groups varied by age	(21)
Marra et al.	2008	Canada, Contacts (mix of foreign born/Canadian born/Aboriginal)	Age Weighted (16–35, 36–55, >56)	(22)
Oxlade et al.	2007	Canada, Migrants or contacts	35	(23)
Pareek et al.	2011	UK, Migrants	35 yrs and younger	(24)
Pooran et al.	2010	UK, Contacts	Not specified	(25)

### Study Quality

For all studies included in the review, the average proportion of “Yes” values given on the quality checklist was 72%. The breakdown of Yes or No/Not clear for each of the 35 checklist items is summarized in Figure S2. The following items from the checklist had the lowest scores across studies; 1) Item 7: The choice of form of economic evaluation is justified in relation to the questions addressed (8% Yes), 2) Item 10: Details of the methods of synthesis or meta-analysis of input values are given (if based on a synthesis of a number of studies) (27% Yes), 3) Item 13: Details of the subjects from whom valuations were obtained were given (33% Yes), 4) Item 14: Productivity changes (if included) are reported separately (25% Yes), 5) Item 27: The choice of variables for sensitivity analysis is justified (38% Yes). A comparison of the text result and abstract conclusion showed discordance in many studies (Summarized in Table S3). The result provided in the text and abstract conclusions on cost effectiveness were totally consistent in only 5 studies.

### Variability of Inputs

Key epidemiologic model inputs reported in studies varied extensively, as shown in Table 1. More detail on these inputs is provided in Table S4. Even after adjustment for inflation and currency, cost inputs included in studies also varied widely (Table 2). For example, the test cost for TST varied from $17 to $121 (2011 USD) and for IGRA, from $21 to $219 (2011 USD). An in depth examination of costing components of these parameters (Table S5) showed that the approaches to costing were different in all studies. For example, 6 studies included costs from the patient perspective, 9 explicitly stated that they included “indirect” or costs for medical staff time to conduct the tests, and 7 explicitly stated that blood draw/phlebotomy costs associated with the IGRA were included.

### Variability of Predicted Results

In all studies, predicted effectiveness measures (QALYs gained or active cases prevented) were almost identical with all test scenarios (Table 4). QALYs gained from use of IGRA relative to use of TST are also shown as ***days*** of life gained, to emphasize the very small differences in effectiveness. Of all studies that compared effectiveness with use of IGRA versus TST, only one study predicted a gain of more than 1 day with use of the IGRA over an analytic horizon of 20 years of more. On the other hand, predicted cost differences between use of IGRA and TST varied widely between studies, and between sub-populations considered within the same study (Table 5).

**Table 4:** Predicted Effectiveness by Screening Strategy.

Study Author, Year	Effectiveness Measure	Population/BCGvaccination status	Effectivenesswith TST	Effectiveness with IGRA	Gain in effectiveness using IGRA (vs TST)
life time analytic horizon					
De Perio, 2009	QALYs	BCG –ve	23.55657	23.55671	0.00014 QALYs (0.05 **days**)
	QALYs	BCG +ve	23.55751	23.55826	0.00075 QALYs (0.27 days)
Deuffic-Brown, 2010	Life expectancy	BCG +ve	25.072	25.073	0.001 Yrs (0.37 days)
Kowada, 2010	QALYs	BCG –ve	22.98153	23.03499	0.053 QALYs (19.51 days)
	QALYs	BCG +ve	22.98153	23.03499	0.053 QALYs (19.51 days)
Kowada, 2010	QALYs	BCG +ve	NA	14.6516	NA
Kowada, 2008	QALYs	BCG +ve	28.1079	28.1099	0.002 QALYs (0.73 days)
Linas, 2011	Life expectancy	Close contacts	23.43917	23.44	0.00083 Yrs (0.30 days)
	Life expectancy	Recent immigrant	25.6925	25.6925	0 Yrs (0 days)
20 year analytic horizon					
Burgos, 2009	QALYs	BCG +ve	NA	11.99	NA
	Active cases	BCG +ve	NA	0.177	NA
Diel, 2007	Active cases*	Mostly BCG+ve	0.0058	0.0058	0 Cases prevented
Diel, 2007	Active cases	Mostly BCG+ve	0.0158	0.0196	−0.018 Cases prevented**
Marra, 2008	QALYs	Foreign born BCG –ve	15.1141	15.1145	0.0004 QALYs (0.15 days)
	QALYs	Foreign born BCG +ve	15.1203	15.1206	0.0003 QALYs (0.11 days)
	Active cases	Foreign born BCG –ve	0.0127	0.0126	0.0001 Cases prevented
	Active cases	Foreign born BCG +ve	0.0064	0.0063	0.0001 Cases prevented
Oxlade, 2007	Active cases	BCG +ve or BCG −ve	0.085	0.085	0 Cases prevented
Pareek, 2011	Active cases	BCG not specified	NA	0.00834	NA
2 year analytic horizon					
Pooran, 2010	Active cases	BCG not specified	0.00452	0.0038 (TSPOT)/0.00403 (QFN)	0.00072 Cases prevented (TSPOT)/0.00049 Cases prevented QFN)

* TB cases predicted in test positive in absence of intervention (treatment).

** Negative sign indicates more cases predicted with IGRA strategy relative to TST.

**Table 5:** Predicted Total Cost per person in 2011 USD by Screening Strategy.

Study Author, Year	Population	TST	IGRA	Cost difference (IGRA vs TST)*
life time analytic horizon				
de Perio, 2009	BCG –ve	$280	$262	-$18
	BCG +ve	$287	$177	-$110
Deuffic-Brown, 2010	BCG +ve	$805	$703	-$102
Kowada, 2010	BCG –ve	$1,920	$1,099	-$821
	BCG +ve	$2,206	$1,099	-$1,107
Kowada, 2010	BCG +ve	NA	$551	NA
Kowada, 2008	BCG +ve	$625	$513	-$112
Linas, 2011	Close contacts	$125, 610	$125, 620	$10
	Recent immigrant	$122,700	$122,700	$0
20 year analytic horizon				
Burgos, 2009	BCG +ve	No data on total cost	No data on total cost	NA
Diel, 2007	BCG +ve	$342	$271	-$71
Diel, 2007	Mostly BCG+ve	$1,376	$748	-$628
Marra, 2008	Mostly BCG+ve	$495	$525	$30
	Foreign born BCG −ve	$460	$452	-$8
Oxlade, 2007	Foreign born BCG – ve	$307	$348	$41
	Foreign born BCG +ve (infancy)	$321	$348	$27
	Foreign born BCG +ve (older)	$382	$348	-$34
Pareek, 2011	BCG not specified	NA	$142	NA
2 year analytic horizon				
Pooran, 2010	BCG not specified	$327	$371 (Tspot)/$369 (QFN)	$295 (TST/TSpot) $285 (TST/QFN)

* A negative number represents a savings with IGRA relative to TST.

### Assessment of Impact of Variability of Study Inputs using a Common Model

**Figure 2:**
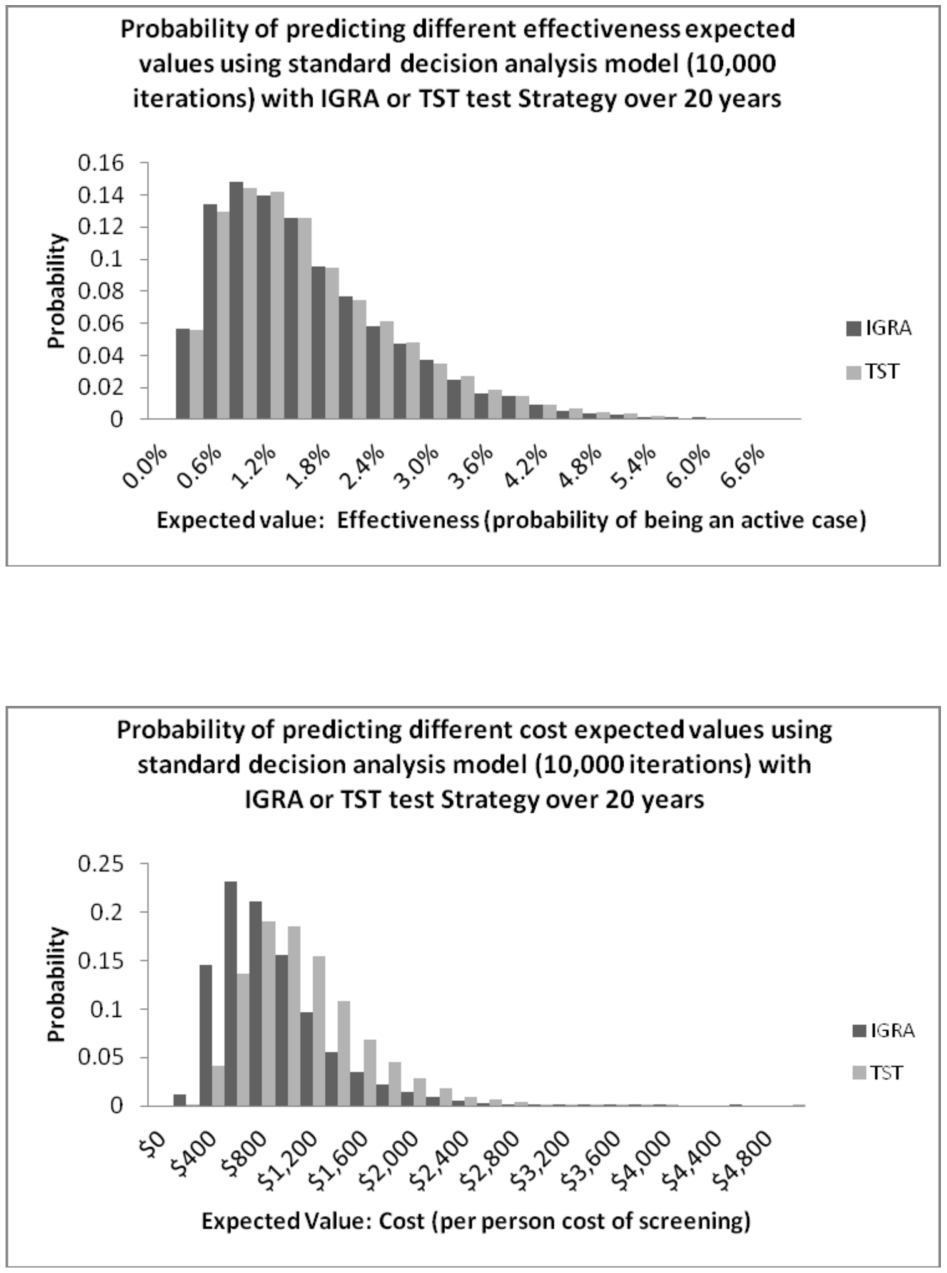
Probability of predicting different expected values using common decision analysis model (10,000 iterations) with IGRA or TST test strategy over 20 years. A. Predicted Effectiveness. B. Predicted Costs.

The distribution of predicted effectiveness (Figure 2a) and costs (Figure 2b) largely overlapped when these outcomes were predicted from Monte Carlo simulations using our common decision analysis model. The 2.5^th^ and 97.5^th^ percentiles for predicted effectiveness (probability of being an active case) were very similar at 0.246% and 4.07% for the TST strategy and 0.244% and 3.98% for the IGRA strategy. Predicted costs showed more of a difference between strategies with the 2.5^th^ and 97.5^th^ percentiles for the TST strategy at $398 and $2251 and for the IGRA strategy at $279 and $1953.

Using the same model, when the model inputs were varied in one way sensitivity analyses, the predicted spread of costs was large (Table 6). For both strategies the parameter with the greatest spread, and thus the greatest potential influence in the model, was the “prevalence of LTBI” (Potential influence: 147% and 97% for IGRA and TST respectively). The reactivation rate in the absence of effective LTBI therapy was also important (Potential Influence: 115% and 92% for IGRA and TST respectively). The cost of treating active TB and LTBI followed in the ranking of influential parameters. The full ranking of all parameters is reported in Table 6.

**Table 6:** One way sensitivity analysis and justification of variability for each input variable included in standard decision analysis model.

Input	Range of input values for each variable (defined using base case value reported in studies in review)	IGRA strategy (Mean expected value: total cost = $855)		TST strategy (Mean expected value: total cost = $1081)		Comment on justification for the variability of inputs used by the different studies
		SPREAD of expected values (|lowest value- highest value|)	POTENTIAL INFLUENCE (Spread/mean expected value)	SPREAD of expected values (|lowest value- highest value|)	POTENTIAL INFLUENCE (Spread/mean expected value)	
Prevalence of LTBI	5% to 58%	$1,258	147%	$1,053	97%	*Variability in probabilities justified:* Differences can be justified by the consideration of different populations and sub-groups.
Reactivation rate (annual) in the absence of effective LTBI therapy	0.02% to 1.25%	$982	115%	$997	92%	*Variability in probabilities predominantly unjustified:* Differences could be justified based on study population (ie. immunosuppressed, close contacts etc.), however inputs used in 11/13 studies were for the general population. In only 2 studies of close contacts were higher reactivation rates used.
Cost of treating active TB	$5318 to $63120	$903	106%	$916	85%	*Variability in costs predominantly unjustified*: All studies should include similar costing components. Costs should be similar for high income settings. Some variability could be due to economic perspective (eg. Study 2 included indirect costs).
Cost of treating LTBI	$224 to $1577	$375	44%	$704	65%	*Variability in costs partially justified:* All studies should include similar costing components. Costs should be similar as most are high income settings. Duration of prophylactic regimen will result in some justified variability in costing (6 studies assumed 9INH, 5 assumed 6INH and 1 assumed 3HR).
Specificity of TST	15% to 99%	−	−	$635	59%	*Variability in probabilities partially justified:* Differences could be justified based on BCG status of study population. However, within each sub population we should see similar estimates that have been derived from meta analyses.
Probability of an adverse event from LTBI therapy	0 to 18%	$318	37%	$558	52%	*Variability in probabilities predominantly unjustified:* Most studies consider young populations that should experience similar types and rates of adverse events. One study considered an elderly population with justified use of higher rates of adverse events.
Completion rate for LTBI therapy	21% to 100%	$271	32%	$267	25%	*Variability in probabilities partially justified:* Estimates should be similar for studies set in general population. Differences based on duration of regimen justified, yet estimates with same duration should be similar.
Cost of an adverse event	$183 to $14006	$236	28%	$442	41%	*Variability in costs predominantly unjustified:* All studies should include similar events with similar costing components.
Cost of IGRA/TST	$21 to $219/$15 to $121	$198	23%	$106	10%	*Variability in costs predominantly unjustified:* All studies should include similar costing components for basic screening. Costs should be similar for high income settings.
Efficacy of LTBI therapy	65% to 90%	$114	13%	$112	10%	*Variability in probabilities partially justified*: Differences justified for different regimens. However, for each regimen, estimate should be similar and derived from previously published meta analyses.
Sensitivity of IGRA	76% to 99%	$32	4%	−	−	*Variability in probabilities predominantly unjustified:* All studies should use similar estimates derived from previously published meta analyses.
Specificity of IGRA	96% to 100%	$31	4%	−	−	*Variability in probabilities predominantly unjustified:* All studies should use similar estimates derived from previously published meta analyses.
Sensitivity of TST	67% to 99%	−	−	$45	4%	*Variability in probabilities predominantly unjustified:* All studies should include similar estimates that have been derived from meta analyses.

## Discussion

Thirteen cost effectiveness papers were reviewed in our study. Differences in estimated effectiveness were consistently very small in all studies. Although in general quality was deemed to be satisfactory, assumed input costs and transitional probabilities were very inconsistent. As a result, predicted costs and cost-effectiveness varied widely. Although CEAs are supposed to provide objective evidence for decision making, when studies present widely discrepant results they are less useful. A lack of standardization and divergence in CEA methods led to the development of the recommendations set out by the Panel on Cost Effectiveness in Health and Medicine in 1996 [26]. Despite the existence of recommendations such as these, many issues still remain in how CEAs are conducted. Some problems appear to stem from how well authors can implement guidelines in practical terms. However, the appropriateness of guidelines for specific areas of evaluative research is also of some concern.

The detailed review of methods performed in this systematic review identified several specific methodologic issues relating to data analysis, presentation and interpretation of CEA findings. The implications of some of these issues are discussed in more detail below:

### Selection of Study Inputs

The estimates of pathogenetic, cost inputs and test characteristics used in different studies varied widely. Even though these inputs played an important role in determining results, much of the variability in input values was not well justified. As highlighted in the Drummond et al [7] evaluative criteria of economic studies, whenever possible, inputs should be derived from systematic reviews and meta analyses.

### Approach to Costing

The approach to costing, including which specific cost components were included, varied by study; this had an important impact on determining cost effectiveness. The Recommendations of the Panel on Cost effectiveness in Health and Medicine [26] for the ideal approach for costing should be followed whenever possible. However, authors are often faced with practical limitations, and in certain cases may have to prioritize using cost data that are easily obtainable.

### Use of the Effectiveness Measure in Diagnostic Studies

The difference in effectiveness measure between test strategies was so small as to be clinically meaningless. Although cost effectiveness is determined by differences in effectiveness and differences in cost, the latter was identified as the main determinant of study results in this particular area. The QALY is recommended by The Panel on Cost effectiveness in Health and Medicine as the ideal measure of health effectiveness [26]. However this study demonstrates a weakness of using this measure for diagnostic studies. Given that none of the conventional measures of effectiveness were able to capture meaningful differences between the two testing strategies for the detection of latent TB infection, the focus of economic studies in this area should be placed on cost alone.

### Presentation and Interpretation of Cost Effective Data

Issues were identified in the presentation and interpretation of data, with many studies not clearly presenting data on which test was “the most cost effective”. Conclusions that a certain strategy was “cost effective” or “highly cost effective” were frequently not defined, or based on a willingness to pay threshold of $50,000 per QALY gained. This benchmark was developed for evaluation of cost-effectiveness of interventions for end stage renal disease in the US in the 1980s [27], so may not be appropriate for the testing scenarios, countries, or populations being considered.

Some of these findings are consistent with the assessment of conceptual issues related to modeling and economic analyses of TB diagnostics by Dowdy et al. [28]. Although they did not focus on IGRAs for diagnosing LTBI, they suggested that current approaches to economic analyses in diagnostic research need to be improved, particularly the need for better defined thresholds for cost effectiveness.

Nienhaus et al. also recently performed a systematic review of TB screening strategies [29]. Unlike our study, their objective was to summarize the evidence in order to make a recommendation regarding a preferred strategy for LTBI screening. Although the authors acknowledged differences in input costs, model assumptions, strategies evaluated and outcomes, they still recommended a preferred test strategy, but cautioned that more evidence is needed for “generally accepted inputs for economic analysis”.

Cost effectiveness analyses are an essential tool for the evaluation of health care practises, and are being used more and more widely to prioritize interventions. Our analysis highlights some of the specific methodological issues observed in published CEA of IGRA screening. Although guidelines exist to standardize such analyses, in many cases these guidelines were not followed, although some aspects may not be relevant for diagnostic CEA. More specific and relevant guidelines are needed, and we suggest the following: 1. The development of standard inputs and assumptions for use in modeling studies like those included in our review would be useful. Standard sources could then be routinely used as input data for modelling studies. 2. The standardization of approaches to costing should also be encouraged so that all studies include similar cost components- ideally from a societal perspective which includes the economic impact on patients in addition to the impact on the health system. 3. The choice of primary economic measure also needs to be considered carefully in these types of studies. Based on our finding of no substantial difference in effectiveness between testing strategies, for this question – of comparing diagnostic strategies in LTBI - economic analyses should focus exclusively on cost and resource implications in the setting in question. 4. Finally, authors should make much greater effort to present and interpret cost effectiveness results in a more transparent manner. For example, standard criteria of willingness to pay must be used and the setting clearly stated when concluding if a study is “cost-effective”. And, if the difference in effectiveness is very small this should be explicitly stated, and any conclusions about cost-effectiveness should be avoided. Ultimately, these recommendations should improve economic studies that evaluate diagnostic strategies for LTBI, and increase their value for informing individual and public health decisions.

## Supporting Information

Figure S1 Simplified Image of Common State-Transition Markov Decision Analysis model.
(DOC)

Figure S2 Bar Graph of Results of Methodologic Quality Assessment by Checklist item.
(DOC)

Figure S3 Drummond 35 Item Quality Checklist.
(DOC)

Table S1 Sample Search String for Medline.
(DOC)

Table S2 Data Abstraction Form.
(DOC)

Table S3 Conclusions of abstracts vs. results reported in text of studies included in review.
(DOC)

Table S4 Epidemiologic input table- Full detail.
(DOC)

Table S5 Costs components (including both Direct costs and Time/Salary labor costs) included by study.
(DOC)
