# Supplementary material for: How Methodologic Differences Affect Results of Economic Analyses: A Systematic Review of Interferon Gamma Release Assays for the Diagnosis of LTBI
Source: PLoS One. 2013 Mar 7;8(3):e56044. doi: 10.1371/journal.pone.0056044 (PMC3591384; doi:10.1371/journal.pone.0056044)
Supplement: Figure S1 — Simplified Image of Common State-Transition Markov Decision Analysis model. (DOC) [file pone.0056044.s001.doc]

Figure S1: Simplified Image of Common State-Transition Markov Decision Analysis model.

Model outlines first year of screening process using either TST test strategy or IGRA test strategy. (See main text for more detail).


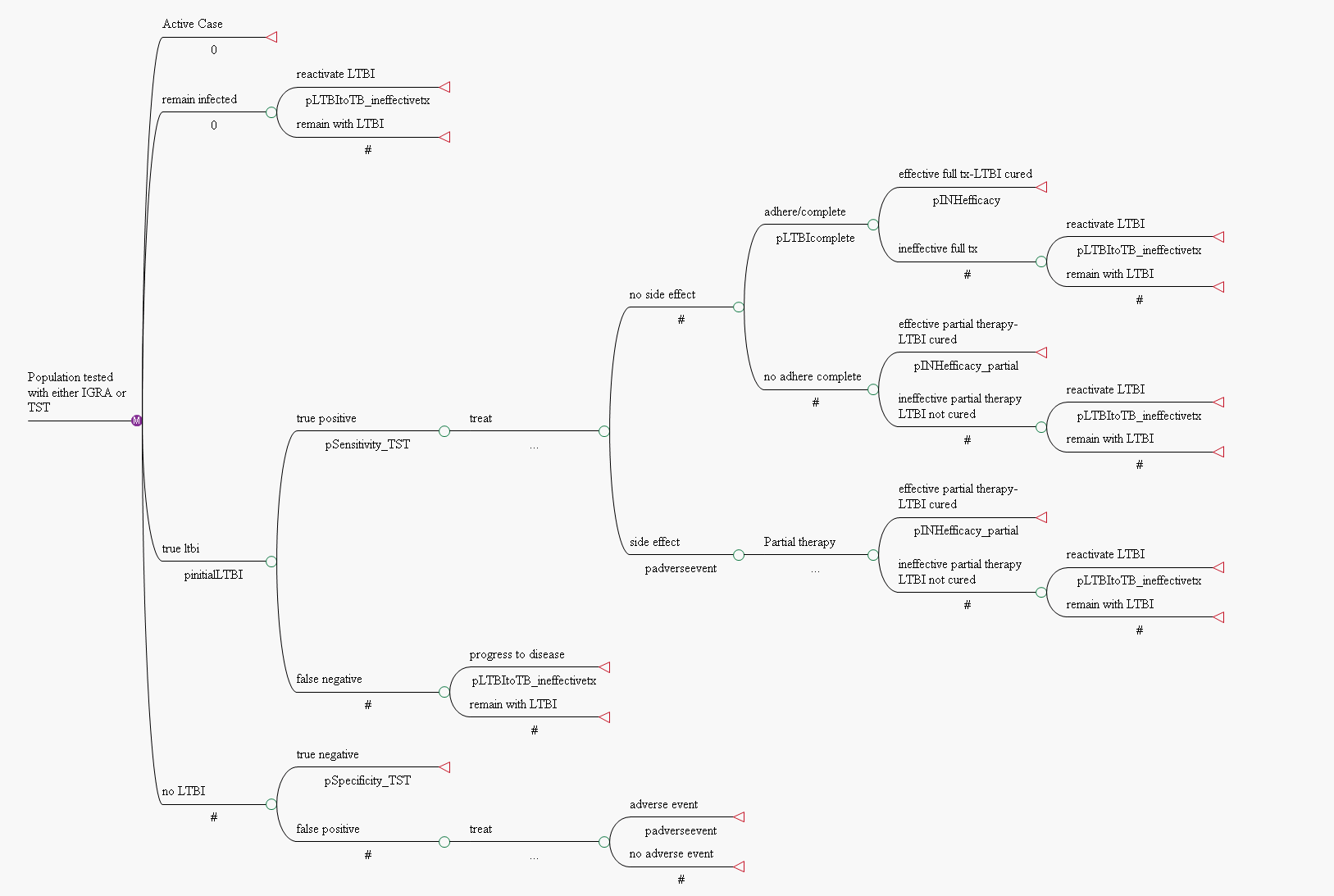


No further consequence

Active case

Cycle to “remain infected”

No further consequence

No further consequence

No further consequence

No consequence

Cycle to “remain infected”

Active case

No further consequence

Cycle to “remain infected”

Active case

Active case

Cycle to “remain infected”

Active case

Cycle to “remain infected”

Cycle to “remain infected”

Active case

Active case

Active case

Cycle to “remain infected”

Cycle to “remain infected”

Cycle to “remain infected”

Active case

Active case

Active case

No further consequence

No consequence

Cycle to “remain infected”

Cycle to “remain infected”

Cycle to “remain infected”

No further consequence
