# Supplementary material for: How Methodologic Differences Affect Results of Economic Analyses: A Systematic Review of Interferon Gamma Release Assays for the Diagnosis of LTBI
Source: PLoS One. 2013 Mar 7;8(3):e56044. doi: 10.1371/journal.pone.0056044 (PMC3591384; doi:10.1371/journal.pone.0056044)
Supplement: Figure S2 — Bar Graph of Results of Methodologic Quality Assessment by Checklist item. (DOC) [file pone.0056044.s002.doc]

Figure S2: Bar graph of Results of Methodologic Quality Assessment by Checklist item (full checklist with description of each item provided below)

* “Yes” and “No” responses are adjusted by the number of responses that were “Not Applicable”

**“No” category plotted in graph includes both “No” and “Not Clear” item responses
