# Supplementary material for: How Methodologic Differences Affect Results of Economic Analyses: A Systematic Review of Interferon Gamma Release Assays for the Diagnosis of LTBI
Source: PLoS One. 2013 Mar 7;8(3):e56044. doi: 10.1371/journal.pone.0056044 (PMC3591384; doi:10.1371/journal.pone.0056044)
Supplement: Table S1 — Sample Search String for Medline. (DOC) [file pone.0056044.s004.doc]

Table S1: Sample Search String for Medline:

Run in Medline: *MEDLINE_R_ In-Process _ Other Non-Indexed Citations March 14_ 2011_ _Database Field Guide_ Ovid MEDLINE_R_ 1948 to March Week 1 2011*

*LIMITS IMPOSED: human only, journal articles only*

| *Mycobacterium Tuberculosis*/ OR Tuberculosis/  AND |
| --- |
| Latent Tuberculosis/ OR Latent.tw OR Dormant.tw OR LTBI.tw OR infection*.tw  AND |
| Diagnosis/ OR detect*.tw OR screen*.tw OR diagnostic test* .tw OR case finding.tw OR case detection .tw  AND |
| tuberculin test / OR TST.tw OR Tuberculin.tw OR skin test*.tw OR PPD.tw OR T-cell-based assay*.tw OR antigen-specific T cell*.tw OR  T cell response*.tw OR T-cell response*.tw OR interferon-gamma / OR interferon*.tw OR IFN .tw OR elispot.tw OR ESAT-6 .tw OR  CFP-10.tw OR culture filtrate protein* .tw OR Enzyme Linked Immunosorbent Spot .tw OR Quantiferon* .tw  AND |
| “Cost and Cost Analysis”/ OR cost-benefit analysis / OR Models, Economic / OR Mathematical model / OR Medical decision making/ OR  Decision making / OR cost*.tw OR economic analys*.tw OR decision analys*.tw OR mathematical model.tw OR modelling stud*.tw OR  modeling stud*.tw |
