# Supplementary material for: How Methodologic Differences Affect Results of Economic Analyses: A Systematic Review of Interferon Gamma Release Assays for the Diagnosis of LTBI
Source: PLoS One. 2013 Mar 7;8(3):e56044. doi: 10.1371/journal.pone.0056044 (PMC3591384; doi:10.1371/journal.pone.0056044)
Supplement: Table S2 — Data Abstraction Form. (DOC) [file pone.0056044.s005.doc]

Table S2: Data Abstraction Form:

Section A- General Information

|  |
| --- |
| Article title: |
| Study number: |
| Authors: |
| Journal : |
| Publication year: |
| Corresponding author country: |
| Commercial interest in topic of paper: Yes No  Declared Undeclared |

| Data extracted by: |
| --- |
| Date completed: |
| Author Contacted: |

| Final Status: |
| --- |
| Notes: |

| Study Design:   - Cost Effectiveness Analysis - Cost Utility Analysis - Cost Benefit Analysis - Budget impact analysis - Other |
| --- |
| Study Population:   - General population - Migrant population (immigration screening) - Health care workers - Arthritis patients - Elderly - Contacts - HIV - Combination of populations-Specify:____________________ - Other |
| Specific age groups considered? Specify:____________________ |
| Tests evaluated – List  Base case:  Strategy 1:  Strategy 2:  Strategy 3:  Strategy 4:  Strategy 5:  Strategy 6:  Additional strategies:________________________________ |
| Author Abstract Conclusion (simplified): |

Section B- Model/ Economic Inputs and Assumptions

| Model detail: |
| --- |
| - Model type: static dynamic |
| - Transmission explicitly included: Yes No - Assumptions made about secondary transmission? Yes No   Specify detail:___________________________ |
| - Type of software used :_____________________ |
| - Analytic horizon: _______________ Cycle length:_________________ |
| Uncertainty:   - Was sensitivity analysis done? Yes No - How were parameters identified?   -no explanation given  -all parameters varied  -justification provided   - How were ranges specified?   -no explanation given  -Review of lit  -Expert opinion  -Using confidence interval (stochastic data)  -other justification   - Was an appropriate form of SA used? - None - One way specific parameters varied - Two way - Multi-way - Scenario analysis- (best case/worst case) - Threshold analysis - Probabilistic/Monte Carlo simulations |

| Economic detail: |
| --- |
| - Perspective (if stated):_________________________ |
| - Types of costs included :   - Government   - health system   - Patient out of pocket   - Patient time/ loss of productivity- cost   - Patient death-cost |
| - Price year :______________ |
| - Currency used :____________________ |
| - Inflation rate Specified: Yes No   Rate:____________ |
| - Discounting:   Were future Costs Discounted ?: Yes No  Were future effectiveness Measure Discounted: Yes No  Rate used:______________ |

| Assumptions: |
| --- |
| 1. Pre test conditions:  - risk of disease   -reactivation rate:____________________________ OR Not Specified  -progression to disease rate:____________________ OR Not Specified   - Mortality of untreated active disease:________________ OR Not Specified - initial prevalence of LTBI:_________________ OR Not Specified - initial prevalence of active disease:__________________ OR Not Specified - initial prevalence of risk factor (if applicable) OR Not Specified - impact of risk factor on disease (RR) (if applicable) :_________OR Not Specified - inclusion of drug resistance? Yes No   - Prevalence of MDR ______________________   - Prevalence of mono-INH resistance ________________ - Inclusion of HIV? Yes No   -Assumptions:____________________   - Utility Weight______________ |
| 1. Test Characteristics  - Test cut offs: - TST _________ - IGRA_________ - T-spot_________ - How was BCG handled? - Age given BCG taken into account? Yes No - TST Test cost (total)_____________ IGRA Test cost (total)_______________ - Includes: Includes: - Labor Labor - Materials Materials - Consumables Consumables  | TEST | Sensitivity | Specificity | Comments | | --- | --- | --- | --- | |  |  |  |  | |  |  |  |  | |  |  |  |  | |  |  |  |  |  - Lab Lab - Equipment Equipment - Transport transport - other____________ other____________   Sensitivity and Specificity: |
| 1. Post test characteristics:  - Medical work up cost: TST:____________ IGRA:______________ - Initial testing drop out included? Yes No - Physician non compliance modelled? Yes No - Patient treatment adherence considered? Yes No - Completion Rate %: _________ - Efficacy of 9 INH:________ - LTBI treatment cost (total):____________ - Inclusion of adverse event costs? Yes No - Type of event:_______________   - Cost of events_____________   - Probability of events_____________ - active TB cost (total):_______________ |

Section C- Data Sources

|  | published literature-systematic review/meta analysis | published literature-RCT | published literature-other (include WHO reports) | unpublished-primary data collection by authors | expert opinion | assumption | other |
| --- | --- | --- | --- | --- | --- | --- | --- |
| Pathogenetic/Epidemiologic |  |  |  |  |  |  |  |
| Test Characteristics |  |  |  |  |  |  |  |
| Effectiveness Measures |  |  |  |  |  |  |  |
| Costs |  |  |  |  |  |  |  |

Section D- Outcomes

|  | BaseCase | Strategy 1 | Strategy 2 | Strategy 3 | Strategy 4 |
| --- | --- | --- | --- | --- | --- |
| Cost per LTBI treated |  |  |  |  |  |
| # active cases |  |  |  |  |  |
| Deaths |  |  |  |  |  |
| QALYs |  |  |  |  |  |
| Life Years |  |  |  |  |  |
| Total Cost |  |  |  |  |  |
| Cost per QALY/LY gained |  |  |  |  |  |
| Cost per case prevented |  |  |  |  |  |
| Other: |  |  |  |  |  |

| - Details of any sensitivity analyses authors chose to consider. |
| --- |

Section E- Quality of Study (Drummond Checklist)

| **Item** | | **Yes** | **No** | **Not clear** | **Not appropriate** |
| --- | --- | --- | --- | --- | --- |
| **Study design** | |  |  |  |  |
| 1. | The research question is stated. | 🞎 | 🞎 | 🞎 |  |
| 2. | The economic importance of the research question is stated. | 🞎 | 🞎 | 🞎 |  |
| 3. | The viewpoint(s) of the analysis are clearly stated and justified. | 🞎 | 🞎 | 🞎 |  |
| 4. | The rationale for choosing alternative programmes or interventions compared is stated. | 🞎 | 🞎 | 🞎 |  |
| 5. | The alternatives being compared are clearly described. | 🞎 | 🞎 | 🞎 |  |
| 6. | The form of economic evaluation used is stated. | 🞎 | 🞎 | 🞎 |  |
| 7. | The choice of form of economic evaluation is justified in relation to the questions addressed. | 🞎 | 🞎 | 🞎 |  |
| **Data collection** | |  |  |  |  |
| 8. | The source(s) of effectiveness estimates used are stated. | 🞎 | 🞎 | 🞎 |  |
| 9. | Details of the design and results of effectiveness study are given (if based on a single study). | 🞎 | 🞎 | 🞎 | 🞎 |
| 10. | Details of the methods of synthesis or meta-analysis of estimates are given (if based on a synthesis of a number of effectiveness studies). | 🞎 | 🞎 | 🞎 | 🞎 |
| 11. | The primary outcome measure(s) for the economic evaluation are clearly stated. | 🞎 | 🞎 | 🞎 |  |
| 12. | Methods to value benefits are stated. | 🞎 | 🞎 | 🞎 | 🞎 |
| 13. | Details of the subjects from whom valuations were obtained were given. | 🞎 | 🞎 | 🞎 | 🞎 |
| 14. | Productivity changes (if included) are reported separately. | 🞎 | 🞎 | 🞎 | 🞎 |
| 15. | The relevance of productivity changes to the study question is discussed. | 🞎 | 🞎 | 🞎 | 🞎 |
| 16. | Quantities of resource use are reported separately from their unit costs. | 🞎 | 🞎 | 🞎 |  |
| 17. | Methods for the estimation of quantities and unit costs are described. | 🞎 | 🞎 | 🞎 |  |
| 18. | Currency and price data are recorded. | 🞎 | 🞎 | 🞎 |  |
| 19. | Details of currency of price adjustments for inflation or currency conversion are given. | 🞎 | 🞎 | 🞎 |  |
| 20. | Details of any model used are given. | 🞎 | 🞎 | 🞎 | 🞎 |
| 21. | The choice of model used and the key parameters on which it is based are justified. | 🞎 | 🞎 | 🞎 | 🞎 |
| **Analysis and interpretation of results** | |  |  |  |  |
| 22. | Time horizon of costs and benefits is stated. | 🞎 | 🞎 | 🞎 | 🞎 |
| 23. | The discount rate(s) is stated. | 🞎 | 🞎 | 🞎 | 🞎 |
| 24. | The choice of discount rate(s) is justified. | 🞎 | 🞎 | 🞎 | 🞎 |
| 25. | An explanation is given if costs and benefits are not discounted. | 🞎 | 🞎 | 🞎 | 🞎 |
| 26. | Details of statistical tests and confidence intervals are given for stochastic data. | 🞎 | 🞎 | 🞎 | 🞎 |
| 27. | The approach to sensitivity analysis is given. | 🞎 | 🞎 | 🞎 | 🞎 |
| 28. | The choice of variables for sensitivity analysis is justified. | 🞎 | 🞎 | 🞎 | 🞎 |
| 29. | The ranges over which the variables are varied are justified. | 🞎 | 🞎 | 🞎 | 🞎 |
| 30. | Relevant alternatives are compared. | 🞎 | 🞎 | 🞎 | 🞎 |
| 31. | Incremental analysis is reported. | 🞎 | 🞎 | 🞎 | 🞎 |
| 32. | Major outcomes are presented in a disaggregated as well as aggregated form. | 🞎 | 🞎 | 🞎 |  |
| 33. | The answer to the study question is given. | 🞎 | 🞎 | 🞎 |  |
| 34. | Conclusions follow from the data reported. | 🞎 | 🞎 | 🞎 |  |
| 35. | Conclusions are accompanied by the appropriate caveats. | 🞎 | 🞎 | 🞎 |  |
