# Supplementary material for: How Methodologic Differences Affect Results of Economic Analyses: A Systematic Review of Interferon Gamma Release Assays for the Diagnosis of LTBI
Source: PLoS One. 2013 Mar 7;8(3):e56044. doi: 10.1371/journal.pone.0056044 (PMC3591384; doi:10.1371/journal.pone.0056044)
Supplement: Table S3 — Conclusions of abstracts vs. results reported in text of studies included in review. (DOC) [file pone.0056044.s006.doc]

Table S3: Conclusions of abstracts vs. results reported in text of studies included in review

| **Author,**  **Year** | **Abstract conclusions** | **Conclusions of abstract vs. text result**  **(For Basecase scenario)** |
| --- | --- | --- |
| Burgos, 2009 | Title: QFT is cost effective in Mexico  Abstract: Targeted LTBI screening and treatment may be highly cost effective in a more specific setting | - Intervention considered is targeted LTBI screening (using IGRA) and treatment in a very high risk Mexican population - Cost effectiveness is justified based on GDP per capita in Mexico using a Willingness to Pay (WTP) threshold of $10,000   Title conclusion does not accurately report the intervention evaluated, nor the population considered.  Abstract conclusion matches what study evaluated |
| de Perio, 2009 | QFT has better outcomes and lower costs than TST. | - QFT Gold was the most cost effective test strategy - QFT was more effective and less costly than TST   Abstract matches text |
| Deuffic-Burban, 2010 | QFT is more effective and less expensive than TST. | - TST followed by QFT combined scenario was the most cost effective - QFT was more effective and less costly than TST   Abstract does not report the most cost effective scenario |
| Diel, 2007 | QFT, but especially QFT following TST, is highly cost effective. | - TST followed by QFT was the most cost effective - Cost effectiveness was justified based on a Willingness to Pay threshold of $50,000   Abstract does not report that combined scenario is best |
| Diel, 2007 | T-SPOT alone or combined with TST is highly cost effective. | - T-SPOT or TST+T-SPOT scenario was the most cost effective (20 year old scenario) - Cost effectiveness was justified based on a Willingness to Pay threshold of $50,000   Abstract matches text |
| Kowada, 2010 | QFT is more effective and less costly than TST (in Rheumatoid Arthritis patients). | - QFT Gold was the most cost effective test strategy - QFT was less costly and more effective than the TST   Abstract matches text |
| Kowada 2010 | No screening is most cost effective (in elderly). | - QFT was the most cost effective strategy - No screening was the least costly strategy - Cost effectiveness is justified based on Willingness to Pay threshold of $50,000   Abstract does not report the most cost effective scenario |
| Kowada 2008 | QFT is more cost effective than TST or TST-QFT (in contacts). | - QFT was the most cost effective strategy - QFT was the least costly and most effective   Abstract matches text |
| Linas, 2011 | IGRA is more cost effective than TST in close contacts, HIV infected and foreign born. | - TST is the most cost effective strategy for the follow groups:   Close contact child, Close contact adult, HIV infected, Homeless, Injection drug user, Immunosupressive medication, Prisoner, Underweight, Gastrectomy, Silicosis, Diabetes and End stage renal disease.   - QFT is the most cost effective strategy for the follow groups:   Recent Immigrant adult, Foreign born living in the US >5 years, all age groups, Recent immigrant child   - Cost effectiveness is justified based on WTP threshold of $100,000   Abstract does not accurately state the most cost effective strategy by population evaluated |
| Marra, 2008 | Selected use of QFT is cost effective. | - Most cost effective strategy was a combined strategy of QFT for BCG vaccinated contacts and TST of all other patients - Criteria to assess cost effectiveness in absolute terms was not defined   Abstract highlights the “most cost effective test”, however no definition of “cost effectiveness” provided |
| Oxlade, 2007 | Screening is cost effective only if risk of disease is high. QFT following TST is more cost effective than QFT. | - Chest X-ray was the most cost effective strategy for Immigrants - TST was the most cost effective strategy for Close contacts and Casual contacts (regardless of region of origin) when non BCG vaccinated, or vaccinated in infancy - QFT was the most cost effective strategy for those BCG vaccinated at an older age   Abstract does not state the most cost effective strategy by population evaluated |
| Pareek, 2011 | LTBI screening can be cost effective | - Most cost effective group to screen with IGRA (vs. no screen) is 16-35 year olds from countries with TB incidence of 250 per 100,000 population - Criteria to assess cost effectiveness in absolute terms was not defined   Abstract doesn’t contradict conclusion, however no definition of “cost effectiveness” provided |
| Pooran, 2010 | A dual testing strategy (T-Spot & TST) is more cost-effective than a single test strategy. | - TST followed by T-SPOT strategy is the most cost effective strategy   Abstract matches text |
