# Supplementary material for: How Methodologic Differences Affect Results of Economic Analyses: A Systematic Review of Interferon Gamma Release Assays for the Diagnosis of LTBI
Source: PLoS One. 2013 Mar 7;8(3):e56044. doi: 10.1371/journal.pone.0056044 (PMC3591384; doi:10.1371/journal.pone.0056044)
Supplement: Table S4 — Epidemiologic input table- Full detail. (DOC) [file pone.0056044.s007.doc]

Table S4: Epidemiologic input table- Full detail.

| **Author,**  **Year** | **Initial Prevalence of LTBI** | **Comment** | **Reactivation rate/yr** | **Comment** | **TST Sensitivity** | **Comment** | **TST Specificity: NO BCG** | **Comment** | **TST Specificity:BCG** | **Comment** | **IGRA Sensitivity** | **Comment** | **IGRA Specificity** | **Comment** |
| --- | --- | --- | --- | --- | --- | --- | --- | --- | --- | --- | --- | --- | --- | --- |
| Burgos, 2009 | 58% | 56%  HIV-ve, 2.3%  HIV +ve | 1.10% | “progression to disease” | NA |  | NA |  | NA |  | 95% | Quantiferon Gold In Tube | 98% |  |
| de Perio, 2009 | 5% |  | 0.02% | Lifetime value (1%) divided by 20 years | 67% | First in a two step | 98% | First in a two step | 70% | First in a two step | 76% | Estimate for Quantiferon Gold In Tube | 96% | Estimate for BCG population (non BCG =100%) |
| Deuffic-Burban, 2010 | 41% | 5% | 0.24% | Reactivation Rate for > 5 years following initial infection | 73% | For 5mm | NA |  | 60% | For 5mm  Data taken from BCG vaccinated populations | 76% | Quantiferon Gold | 96% |  |
| Diel, 2007 | 11% | Based on True positives (100) and False negatives (10) predicted with QFT G/TST 5mm | 0.30% | Reactivation rate For 5mm TST | 90% | For 5 mm | NA | For 5mm | 61% |  | 90% | Quantiferon Gold | 100% |  |
| Diel, 2007 | 28% | Based on T spot positive | 0.30% | Reactivation rate For 5mm TST | 93% | For 5mm | NA |  | 25% |  | 95% | T SPOT | 100% |  |
| Kowada, 2010 | 5% | Info from author- not in publication | 0.28% | Rate for middle age group | 80% |  | 97% |  | 59% |  | 84% | Quantiferon | 99% |  |
| Kowada, 2010 | 36% |  | 0.15% | Rate for middle age group | NA |  | NA |  | NA |  | 81% | Quantiferon | 99% |  |
| Kowada, 2008 | 20% | “chance of developing TB/LTBI” | 0.28% |  | 71% | For 10mm | 98% |  | 15% | 15% given as “basecase”. 60% given for BCG vaccinated in older children | 76% | Quantiferon | 96% |  |
| Linas, 2011 (adult close contacts) | 43.7% | Estimate for adult close contacts | 1.02% | Rate cited for adult close contacts | 89% |  | 98% | Estimate for US born | NA |  | 83% | Generic IGRA | 99% |  |
| Linas, 2011(recent adult immigrants to US) | 41.4% | Estimate for recent adult immigrants | 0.08% | Rate cited for recent adult immigrants | 89% |  | NA | NA | 92% | Estimate for foreign born | 83% | Generic IGRA | 99% |  |
| Marra, 2008 | 21% | Differs by populat ion. Estimate shown for foreign born | 0.41% |  | 99% |  | 95% | Estimate taken from BCG –ve Foreign born population | 36% | Estimate taken from BCG +ve  Foreign born population | 99% | Quantiferon Gold | 96% |  |
| Oxlade, 2007 | 35% | Differs by population. Estimate shown for immigrants from intermediate incidence | 0.10% |  | 95% |  | 98% |  | 60% | Estimate for vaccination in older children | 95% | Quantiferon Gold | 98% |  |
| Pareek, 2011 | 23% |  | 0.25% | Assumed 5% over 20 years, giving 0.25%/yr | NA |  | NA |  | NA |  | 84% | Quantiferon Gold In Tube | 99% |  |
| Pooran, 2010 | 30% |  | 1.25% | 2.5% over 2 years, | 85% |  | 80%*  No mention of BCG |  | NA |  | 95/89 | T Spot/  IGRA | 100/95 | Tspot/  IGRA |

Note: If study considered contacts restricted to 5mm estimate for TST sensitivity & specificity. If study considered health care worker, immunosuppresed or elderly populations used 10mm estimate for TST sensitivity & specificity.

Table S4 (Continued): Epidemiologic input table- Full detail

| **Author,**  **Year** | **LTBI Adherence/Completion rate** | **Comment** | **Efficacy of LTBI regimen** | **Comment** | **Probability of adverse event** | **Comment** |
| --- | --- | --- | --- | --- | --- | --- |
| Burgos, 2009 | 80% |  | 69% | 6 month regimen | 18% | INH toxicity |
| de Perio, 2009 | 31% | 96% prescribed, 68% accepted, 48% finished | 90% | 9 month regimen | 0.60% | Severe hepatitis |
| Deuffic-Burban, 2010 | 57% |  | 69% | 9 month regimen | 0.001% | Severe toxicity |
| Diel, 2007 | 100% | No information provided on compliance. | 80% | 9 month  regimen | 0% | Side effects from INH are not considered |
| Diel, 2007 | 100% | Assumed all patients take a full course of therapy | 80% | 9 month  regimen | 0% | Side effects from INH are not considered |
| Kowada, 2010 | 80% |  | 70% | 6 month regimen | 0.30% | Hepatitis |
| Kowada, 2010 | 80% |  | 70% | 6 month regimen | 0.30% | Hepatitis |
| Kowada, 2008 | 90% |  | 70% | 6 month regimen | 1.30% |  |
| Linas, 2011  (adult close contacts) | 48% | Assumes table 1 is overall completion (includes 80% return to read and 90% start) | 90% | 9 month regimen | 1% | Severe Hepatitis - age >35 |
| Linas, 2011  (recent adult immigrants to US) | 51% | Assumes table 1 is overall completion (includes 80% return to read and 90% start) | 90% | 9 month regimen | 1% | Severe Hepatitis - age >35 |
| Marra, 2008 | 28% | 8% don’t return for 1^st^ test, 61% probability starting if eligible. 50% complete | 90% | 9 month regimen | 0.30% | Hepatitis |
| Oxlade, 2007 | 21% |  | 80% | 9 month regimen | 1% | Hepatitis |
| Pareek, 2011 | 81% | 95% accept, 85% complete | 65% | 3 month regimen | 0.20% | Hepatitis |
| Pooran, 2010 | 72% | 90% return, 80% start | 65% | 6 month regimen | 0.3% |  |
