# Supplementary material for: How Methodologic Differences Affect Results of Economic Analyses: A Systematic Review of Interferon Gamma Release Assays for the Diagnosis of LTBI
Source: PLoS One. 2013 Mar 7;8(3):e56044. doi: 10.1371/journal.pone.0056044 (PMC3591384; doi:10.1371/journal.pone.0056044)
Supplement: Table S5 — Costs components (including both Direct costs and Time/Salary labor costs) included by study. (DOC) [file pone.0056044.s008.doc]

Table S5 Costs components (including both Direct costs and Time/Salary labor costs) included by study

| **Author,**  **Year** | **TST** | | | **IGRA** | | |
| --- | --- | --- | --- | --- | --- | --- |
|  | **Total cost reported**  **(USD 2011)** | **Direct Cost Components** | **Time/ Salary related Cost Components**** | **Total cost reported**  **(USD 2011)** | **Direct Cost Components** | **Time/ Salary related Cost Components**** |
| Burgos, 2009 | No TST | N/A | N/A | 20.68 | -Test and supplies | None explicitly reported |
| de Perio, 2009 | 59.86 | -2 step Test and supplies* | -Medical Staff time (nurse for place and read and clerk)  -Health care worker time lost from work | 49.14 | -Test and supplies  -laboratory supplies  -phlebotomy supplies | -Medical Staff time (nurse, phlebotomist, laboratory scientist, clerk)  -Health care worker time lost from work |
| Deuffic-Burban, 2010 | 17.25 | -Test | -Two health system visits(hospital) | 71.22 | -Test | -One health system visit (hospital) |
| Diel, 2007 | 32.34  Detail From Cost Optimization study (Diel ERJ, 2006) | -Test | -Medical staff time for administration of test (Medical technical assistant)  -Medical staff time for read and registration of result (physician) | 79.44 | -Test (QFT tubes)  -Blood draw | -Medical staff time for blood draw (Medical-Technical Assistant)  -Staff time for interpreting test result (unspecified) |
| Diel, 2007 | 38.96 | -Test and supplies | - Medical Staff time (unspecified) | 218.50 | -Test and supplies  -Laboratory fees | -Medical Staff time (physician) |
| Kowada, 2010 | 109.20 | No detail provided | No detail provided | 117.31 | -Test and supplies  -Blood draw | -Medical Staff time (laboratory technician)  - One health system visit (physician) |
| Kowada, 2010 | No TST | N/A | N/A | 92.34 | -Test and supplies  -Blood draw | -Medical Staff time (laboratory technician)  -One health system visit (physician) |
| Kowada, 2008 | 120.92 | -Test and supplies | -Medical Staff time (unspecified)  -Two health system visit (physician) | 118.23 | -Blood draw  -Test and supplies | -Medical Staff time (laboratory technician)  -One health system visit (physician) |
| Linas, 2011 | 41.80 | -Test | -Medical Staff time (Nurse)  -Medical Staff time for test reading (unspecified) | 51.77 | Detail not provided in paper. Cost based on other cost-effectiveness analysis. | |
| Marra, 2008 | 27.88 | -Test and supplies  -Equipment | -Medical Staff time for inject and read (unspecified) | 49.73 | -Test and supplies  -Equipment | -Medical Staff time (unspecified) |
| Oxlade, 2007 | 14.62 | -Test supplies | -Medical Staff time (unspecified) | 47.46 | -Test and supplies  -Transportation  -Reporting | -Medical Staff time (unspecified ) |
| Pareek, 2011 | No TST | N/A | N/A | 77.43 | -Test and supplies  -Blood draw | -None explicitly reported |
| Pooran, 2010 | 29.34 | -Test and supplies | - Medical Staff time for administration and reading (unspecified) | 81.79 (QFT); 99.97 (Tspot) | -Test and supplies  -Blood draw  -Test processing | - None explicitly reported |

*2 step TST- took into account both tests, weighted by probability of being placed and read

** Includes Time and Salary labor costs associated with any type of “health system visit”
